# Antiseptic Polymer–Surfactant Complexes with Long-Lasting Activity against SARS-CoV-2

**DOI:** 10.3390/polym14122444

**Published:** 2022-06-16

**Authors:** Vyacheslav S. Molchanov, Andrey V. Shibaev, Eduard V. Karamov, Viktor F. Larichev, Galina V. Kornilaeva, Irina T. Fedyakina, Ali S. Turgiev, Olga E. Philippova, Alexei R. Khokhlov

**Affiliations:** 1 Physics Department, Lomonosov Moscow State University, 119991 Moscow, Russia; molchan@polly.phys.msu.ru (V.S.M.); shibaev@polly.phys.msu.ru (A.V.S.); khokhlov@polly.phys.msu.ru (A.R.K.); 2 Gamaleya National Research Center for Epidemiology and Microbiology of the Russian Ministry of Health, 123098 Moscow, Russia; karamov2004@yandex.ru (E.V.K.); vlaritchev@mail.ru (V.F.L.); kornilaeva@yandex.ru (G.V.K.); irfed2@mail.ru (I.T.F.); turgiev@ld.ru (A.S.T.); 3 National Medical Research Center of Phthisiopulmonology and Infectious Diseases of the Russian Ministry of Health, 127473 Moscow, Russia

**Keywords:** polyelectrolyte, hydrogel, surfactant, COVID-19, SARS-CoV-2, disinfectant, polyacrylamide

## Abstract

Antiseptic polymer gel–surfactant complexes were prepared by incorporating the low-molecular-weight cationic disinfectant cetylpyridinium chloride into the oppositely charged, slightly cross-linked polymer matrices. Three types of polymers were used: copolymers of acrylamide and sodium 2-acrylamido-2-methylpropane sulfonate; copolymers of acrylamide and sodium methacrylate; copolymers of vinylpyrrolidone and sodium methacrylate. It was shown that the rate of the release of the cationic disinfectant from the oppositely charged polymer gels could be tuned in a fairly broad range by varying the concentration of the disinfectant, the degree of swelling, and degree of cross-linking of the gel and the content/type of anionic repeat units in the polymer matrix. Polymer–surfactant complexes were demonstrated to reduce SARS-CoV-2 titer by seven orders of magnitude in as little as 5 s. The complexes retained strong virucidal activity against SARS-CoV-2 for at least one week.

## 1. Introduction

The coronavirus disease (COVID-19), caused by severe acute respiratory syndrome coronavirus 2 (SARS-CoV-2), appeared in China at the end of 2019 [1]. By March 2020, the disease was raging throughout most countries of the world, and the World Health Organization (WHO) declared COVID-19 a pandemic [1,2]. By 24 May 2022, the pandemic had caused more than 6.3 million deaths, according to Worldometers [3].

The fight against the infection is complicated by the high mutation rate of the virus. This requires introducing changes into the composition of the vaccines, which cannot be achieved fast enough. In addition, no specific therapy was proposed that can address all variants and mutants of SARS-CoV-2 at once. These factors make early prevention of the infection crucial to control its spreading. The preventive measures should comprise, in particular, the use of effective and human-friendly antiseptic coatings that restrict contact transmission through surfaces contaminated by respiratory droplets of the infected people [4]. It was reported that, at room temperature, SARS-CoV-2 could persist on different surfaces (such as plastic, stainless steel, metal, and glass) for up to 3–4 days [5,6,7,8]. Moreover, the virus was found on various surfaces in the cabins of the Diamond Princess cruise ship 17 days after infected passengers vacated [9]. Commonly used disinfectants, including alcohols and quaternary ammonium compounds, can efficiently inactivate the virus [10,11,12,13,14,15], but their action does not last long because they are easily washed away [16]. To provide continuous action, they should be reused frequently, which can cause undesirable toxicological effects [2,17,18]. An alternative possibility for ensuring prolonged antiseptic activity would be to use polymers as carriers for disinfectant molecules [2,19]. Polymer matrices can provide a prolonged release of the disinfectant in a controlled fashion [19].

Of particular interest is to use polymer matrices composed of anionic polyelectrolyte gel that is able to form complexes with oppositely charged disinfectants—quaternary ammonium compounds. These compounds are believed to exert antiviral effects by disrupting the protective lipid coating [20,21] of enveloped viruses (all coronaviruses, including SARS-CoV-2, belong to enveloped viruses [22]). The disruption may proceed through adsorption of quaternary ammonium compounds to the negatively charged surface of the virus via electrostatic attraction, followed by the penetration of hydrophobic alkyl chain into the lipid bilayer of the envelope. An intact viral envelope is a prerequisite to virus interaction with its receptors on the target cells, which makes possible penetration therein and initiation of the infection [23]. Thus, envelope perturbation/disruption is a way to inactivate SARS-CoV-2 and prevent infection [24].

Many quaternary ammonium compounds were shown to be quite efficient against SARS-CoV-2; those include cetylpyridinium chloride (CPC) [24,25,26], benzalkonium chloride (BAC) [6,13,15], di-N-didodecyldimethylammonium bromide, and di-N-decyldimethylammonium chloride [14]. For instance, CPC that has already been FDA approved for use in commercial healthcare products [27] demonstrated virucidal activity against SARS-CoV-2 both in vitro [24,25] and in vivo [26]. BAC was used for the fabrication of face masks [28] and face shields [29], capable of inactivating SARS-CoV-2. Note that the above-mentioned quaternary ammonium compounds are cationic surfactants since each contains long hydrophobic tail(s) in addition to a cationic group.

Being introduced into a polymer–gel matrix, the molecules of a disinfectant can be gradually released over extended periods, providing a long-term antiseptic activity [19,30,31,32,33,34]. It was shown that the presence in the gel of units with a charge opposite to that of the disinfectant increases its load significantly and favors prolonged release [30].

Many efforts are directed toward the development of antiseptic systems based on polyelectrolyte gels and oppositely charged disinfectants [19,30,31,32,33,34]. For example, Ref. [19] describes gels based on poly(4-vinylbenzyl chloride-co-acrylic acid) and poly(sodium 4-styrenesulfonate-co-glycidyl methacrylate) copolymers modified by covalent or electrostatic binding of the biocides (4-vinyl benzyl dimethylhexadecylammonium chloride and cetyltrimethylammonium 4-styrenesulfonate, respectively). It was shown [19] that the system with the electrostatically bound cationic surfactant is more active against *S. aureus* and *P. aeruginosa*. In another paper [31], cross-linked multilayer films with a pH-triggered release of an antibacterial agent were prepared from poly(methacrylic acid) and an oppositely charged antimicrobial peptide derived from lactoferrin. In a recent study [34], microgels based on an interpenetrating network of poly-N-isopropylacrylamide/polyacrylic acid, loaded with the oppositely charged disinfectant BAC were elaborated. The microgels show antiseptic efficacy against *Bacillus subtilis* and *S. aureus*, which is approximately equal to that of commercial antibiotics.

Most of the reports on polymer gel/disinfectant systems have focused on antibacterial properties. Studies of antiviral activity of such materials are scarce [35,36], most of them dealing with uncross-linked polyelectrolytes. For instance, in Ref. [37], a water-soluble antiseptic complex of poly(N-vinylpyrrolidone-co-crotonic acid) with BAC was proposed, which was shown to effectively destroy nonenveloped and enveloped viruses (coronaviruses, rotaviruses, adenoviruses, AH1N1 influenza virus). In a recent report [38], a water-insoluble complex of polystyrene sulfonate with BAC exhibiting anti-SARS-CoV-2 activity was elaborated. To the best of our knowledge, no complexes based on cross-linked polyelectrolyte gels were tested for the virucidal activity against SARS-CoV-2. At the same time, cross-linking provides many advantages for producing carriers for antiseptic agents. For instance, cross-linked materials do not lose polymer during use (e.g., as a result of dissolution) and, therefore, can be fully rejuvenated by reloading with the disinfectant.

**Chart 1:**
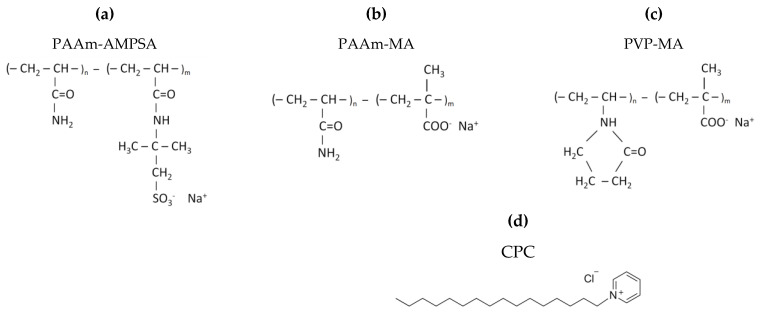
Chemical structures of anionic polyelectrolytes: (**a**) poly(acrylamide-co-sodium 2-acrylamido-2-methylpropane sulfonate) (PAAm-AMPSA), (**b**) poly(acrylamide-co-sodium methacrylate) (PAAm-MA), (**c**) poly(vinylpyrrolidone-co-sodium methacrylate) (PVP-MA), and (**d**) chemical structure of cationic surfactant cetylpyridinium chloride (CPC).

The aim of the present study is to develop antiseptic coatings with long-lasting activity against SARS-CoV-2, which are based on negatively charged polyelectrolyte gels with an embedded model antiseptic—the quaternary ammonium compound CPC [39,40,41] (Chart 1). Three types of negatively charged polyelectrolyte gels were used (Chart 1): poly(acrylamide-co-sodium 2-acrylamido-2-methylpropane sulfonate) (PAAm-AMPSA), poly(acrylamide-co-sodium methacrylate) (PAAm-MA), and poly(vinylpyrrolidone-co-sodium methacrylate) (PVP-MA). Such systems offer promise as coatings for various surfaces in public areas (hospitals, shopping malls, public transport, etc.).

## 2. Materials and Methods

### 2.1. Materials

Acrylamide (AAm) (purity > 98%), 2-acrylamido-2-methyl-1-propanesulfonic acid (AMPSA) (purity > 99%), methacrylic acid (MA) (purity > 99%), ammonium persulfate (purity > 98%), N,N,N’,N’-tetramethylethylenediamine (purity > 99%), 1-vinyl-2-pyrrolidone (VP) (purity > 99%), and CPC (purity > 98%, critical micelle concentration (CMC) in water 0.9 mM [42,43,44]) were provided by Sigma Aldrich (St. Louis, MO, USA). N,N′-methylenebisacrylamide (purity > 99%), azobisisobutyronitrile (purity > 98%), and polyethylene glycol (PEG) with a molar mass of 1000 g/mol were obtained from Fluka (New York, NY, USA). Isopropanol was purchased from Acros Organics (Geel, Belgium). All chemicals were used as received. The solutions were prepared using distilled deionized water obtained by MilliQ system (Millipore, Burlington, MA, USA).

Vero E6 cells (ATCC, Manassas, VA, USA; catalog number CRL-1586) were cultured in high glucose Dulbecco’s modified Eagle’s medium (DMEM; Sigma-Aldrich, St. Louis, MO, USA) supplemented with 5% fetal calf serum (FCS), 2 mM L-glutamine, and antibiotics (150 u/mL penicillin and 150 u/mL streptomycin) at 37 °C in 5% CO_2_.

The stock of SARS-CoV-2 (strain HCoV-19/Russia/Moscow-PMVL-12/2020 (EPI_ISL_572398) isolated from a patient) was the culture liquid withdrawn from cultures of the infected Vero E6 cells.

### 2.2. Preparation of the Hydrogels

Hydrogels were synthesized by free-radical copolymerization of uncharged and charged comonomers and a cross-linker (N,N′-methylenebisacrylamide).

Synthesis of PAAm-AMPSA and PAAm-MA hydrogels was carried out in water. Monomers, cross-linker, initiator (ammonium persulfate), and PEG were dissolved in water in appropriate quantities. After that, the solution was degassed by purging argon for 5 min, and a catalyst (N,N,N′,N′-tetramethylethylenediamine) was added. Polymerization was carried out for 8 h at room temperature.

Synthesis of PVP-MA gels was performed in isopropanol. After the dissolution of monomers, cross-linker, and initiator (azobisisobutyronitrile), polymerization was carried out for 2 h at 70 °C under constant purging of argon through the reaction medium.

After synthesis, the gels were put into a large volume of 10^−4^ M NaOH and then distilled water for 12 h to remove unreacted chemicals; the outer solution was changed a few times. In the case of PEG-containing gels, the outer solution had the same PEG concentration as the gels in order to prevent PEG release from the gel. For PAAm-AMPSA gels, the sol fraction was less than 0.5%, as determined by weighing the dried samples. For PVP-MA gels, the sol fraction was larger (~30%), which may be due to the lower reactivity of VP monomers as compared to other vinyl monomers [45].

The degree of swelling of the gels was determined according to the formula [46]: β = (m_sw_ − m_0_)/m_0_, where m_sw_ is the mass of the swollen gel and m_0_ is the mass of the dry gel.

### 2.3. Loading of the Hydrogels with Disinfectants

**Figure 1:**
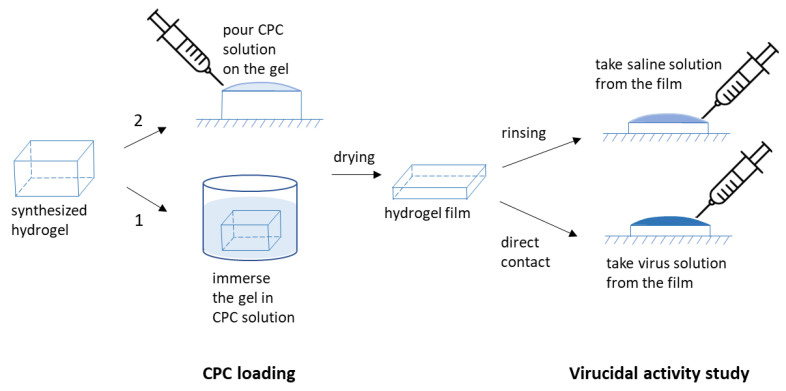
Schematic representation of the experiments on CPC loading and virucidal activity study.

To load the hydrogels with CPC, two methods were used (Figure 1). Method 1: the gels were immersed in an aqueous solution of CPC (the concentration of CPC was equal to 0.028 M or 0.14 M for the gels containing 2 and 10 mol% of charged units, respectively). Method 2: 0.1–0.5 mL 0.244 M aqueous CPC was poured onto the surface of the hydrogel. The hydrogels prepared using methods 1 and 2 were designated by letters v and s, respectively.

Note that, in these experiments, we tried to avoid the formation of dense shells of polymer–surfactant complex, which are observed when the surface region collapses because of complex formation, whereas the central part of the gel remains in the swollen state [47,48,49,50]. Such collapsed shells release a considerable amount of CPC, so that the disinfectant is quickly exhausted, shortening the antiseptic activity of the material. We observed that the formation of collapsed shells could be avoided if the concentration of CPC solution used for loading is rather low (for instance, lower than 0.25 M for PAAm-AMPSA-4s hydrogel).

To reload the hydrogels with CPC after their full consumption, an appropriate volume of solution containing 7 wt% CPC and 25 wt% PEG was poured on their surface.

### 2.4. UV-Spectroscopy

UV-spectroscopy was used to follow the release of CPC from the hydrogel films. The experiments were performed with the SF-2000 spectrophotometer (OKB Spektr, Saint-Petersburg, Russia). CPC concentration was determined from the intensity of the absorbance peak of the pyridinium ring at 259 nm (Figure S1, Supplementary Materials). The intensity–concentration calibration curve was obtained by using CPC water solutions with known concentrations in the range 0.01–0.3 mM (Figure S2, Supplementary Materials). It gave the extinction coefficient of 4070 L/mol·cm, which is consistent with the literature data [43]. The solutions with CPC released from the polymer matrix were diluted 10–200 times with distilled water before the measurements in order to obtain the optical density D in the range from 0.1 to 1.

### 2.5. NMR Spectroscopy

^1^H NMR measurements were carried out on a Bruker AV-600 spectrometer (Billerica, MA, USA) at 30 °C in D_2_O as a solvent. The gel samples were degraded for 10 min by ultrasonic homogenizer Cole-Parmer CPX500 in order to break the gel, and then were put in standard 5 mm NMR tubes (Norell). The acquisition time of 2 s was used and 256 scans were performed. The data were processed using MestReNova software, including baseline, reference, and phase correction. ^1^H chemical shifts were referenced to the HOD signal.

### 2.6. Mechanical Tests

Indentation load–unload tests of the hydrogel coatings were performed on an LS5 testing machine (Lloyd Instruments Ltd., Segensworth, Hampshire, UK) at room temperature. Load–depth curves were obtained on disk-like samples (diameter 30 mm, height 4 mm) with a 5 kN load cell and cylindrical indenter (diameter 8 mm) at the constant load–unload speed of 0.5 mm/min.

### 2.7. Release Studies

To study CPC release from hydrogel samples, two main approaches were used. In the first approach, a small volume of saline solution was poured onto the gel surface. In the second approach, the gels were immersed in a large volume of saline (exceeding about 100 times that of the polymer gel).

### 2.8. Antiviral Activity Tests

#### 2.8.1. Virucidal Activity of CPC Solutions

Solutions of CPC (1 mL) were incubated at room temperature with an equal volume of the virus stock for 1 h. To avoid possible toxic effects of CPC on the cells (Table S1, Supplementary Materials), the samples (CPC + virus) were centrifuged at 27,000 rpm for 1 h. A positive control (the virus stock without CPC) was used in every run. Viral pellets were resuspended in 300 µL of support medium (DMEM, 1% FCS), and serial 10-fold dilutions in support medium were prepared for titer determination. The dilutions thus obtained were used to infect confluent Vero-E6 monolayers in 96-well plates; following 2 h of incubation, the inoculum was removed, and the plates were washed twice with FCS-free DMEM. Thereafter, the plates were filled with another type of support medium (DMEM, 2% FCS) and further incubated at 37 °C in 5% CO_2_ for 96 h.

Virus-induced cytopathic effects (structural changes in the cells caused by the viral invasion) were assessed by microscopic examination of the wells; the percentage of the infected cells was used for virus quantification. The amount of the active virus was determined by endpoint dilution (titration) and expressed in fifty-percent tissue culture infective doses (TCID_50_). The titer was calculated using the Spearman–Kärber method and presented as lg TCID_50_/0.1 mL [51,52].

The virucidal activity of CPC was assessed by the difference in the virus titers (A) between control (A_c_) and experimental (A_e_) samples:

A = A_c_ − A_e_

The protection index, or inhibition coefficient (IC), was calculated using the formula:

IC = [(A_c_ − A_e_)/A_c_] × 100%

#### 2.8.2. Virucidal Activity of CPC Formulated into Gels

Two experimental setups were used to assess the efficacy of CPC formulated into gels (Figure 1).

(i) Washes from the surface of the gel: phosphate-buffered saline, pH 7.2 (450 μL), was applied dropwise onto pieces of CPC-containing gels, and placed in Petri dishes; after 5 s, the flowing liquid was collected and used for incubation with the virus (300 μL) and spectrophotometric measurement of the amount of CPC released from the gels as a result of the washing (residual volume, less than 150 µL). The virucidal activity of CPC washed off the surface of the gels was determined (as described above) 24 h (on day 1) and 168 h (on day 7) after the gels were prepared.
(ii) Direct contact of the virus with the surface of CPC-containing gels: 150 µL of support medium containing the known amount of the virus was applied onto the surface of the gel for 5 s, after which the flowing liquid was collected and the titer of the virus, determined as described above (in order to establish changes in the titer caused by the contact with the surface of the CPC-containing gel).

## 3. Results and Discussion

### 3.1. Preparation and Characterization of Antiseptic Polymer Coatings

**Figure 2:**
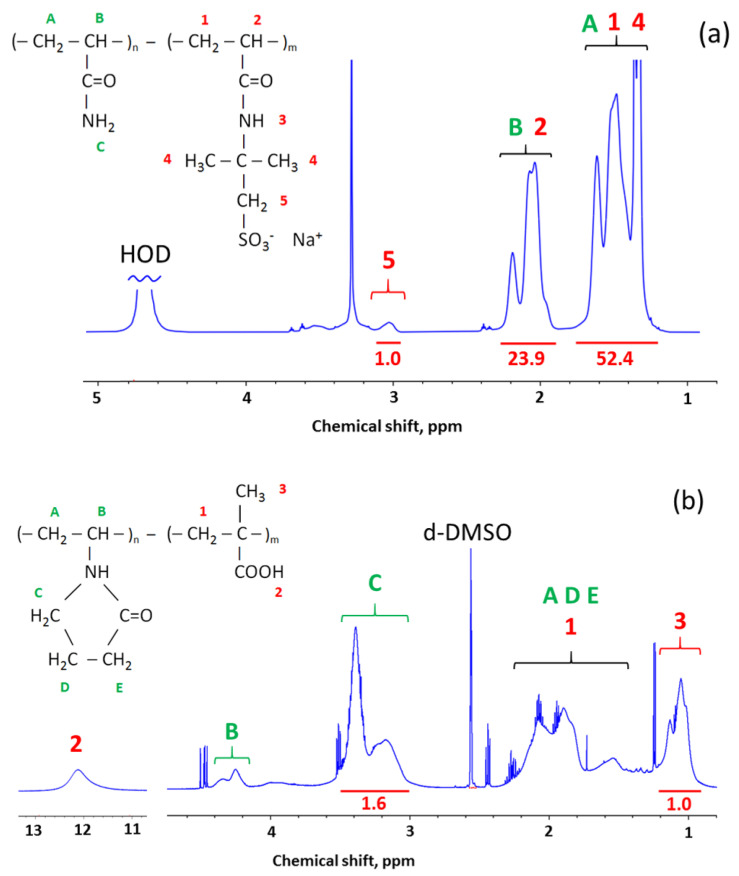
^1^H NMR spectra of synthesized copolymer gels: (**a**) PAAm-AMPSA in D_2_O, (**b**) PVP-MA in d-DMSO. Peak assignments were made according to Refs. [53,54,55]. At the top, the peaks are labeled by letters (A, B, C, etc.) and numbers (1, 2, 3, etc.) corresponding to different protons in the polymers’ chemical structure drawn in the Figure. At the bottom, peak integrals used for quantification of the polymers’ composition are written under horizontal red lines.

To prepare antiseptic polymer coatings, three types of negatively charged copolymer gels were used: PAAm-AMPSA, PAAm-MA, and PVP-MA. They were prepared by free-radical copolymerization of uncharged and charged monomers, using N,N′-methylenebisacrylamide as a cross-linker. The degree of cross-linking was very low (Table 1), which facilitates the formation of coatings from these gels while avoiding the loss of polymer when in contact with water. The composition of the gels was confirmed by ^1^H NMR (Figure 2 and Table 1).

The polymer gels were further modified by electrostatic binding of the cationic surfactant CPC acting as a disinfectant. The disinfectant loading was performed in two ways. In the first way, the whole gel sample was immersed in CPC solution. In the second way, CPC solution was applied only onto one surface of the gel (Figure 1).

The interaction of ionic surfactants with oppositely charged polyelectrolyte gels has been the subject of extensive studies, both experimental [42,43,47,48,49,56,57,58] and theoretical [49,59]. It was shown that surfactant ions penetrate the gel as a result of an ion exchange reaction with polymer counterions. If the initial charge density on the gel subchains is high, the within-the-gel concentration of the surfactant may considerably exceed its concentration in the outer solution. As a consequence, micelle formation will start within the gel in the absence of micelles in the outer solution. Note that the critical association concentration of surfactant within the oppositely charged gel is always 1–2 orders of magnitude lower than the CMC of the same surfactant in water [58]. Theoretically [59], the reason for this phenomenon was understood as described below. When the micelles are formed in the solution, some parts of counterions become condensed near their highly charged surface, losing their entropy of independent translational motion. By contrast, within the gel, the micelle charge is neutralized by ions that are initially immobilized on polymer chains so that no additional loss of translational entropy occurs.

When the amount of the bound surfactant ions is close to that of the charged polymer units, the volume of the initially highly swollen hydrogel decreases considerably, i.e., the gel collapse occurs [60,61]. The collapse is due to the decrease in the osmotic pressure within the gel, resulting from surfactant aggregation into micelles. The collapsed gel can further absorb the surfactant over the equimolar surfactant/gel charged units ratio corresponding to electroneutrality due to hydrophobic interactions. In this case, surfactant ions penetrate the gel together with their counterions, which causes the osmotic pressure to increase, leading to the reswelling of the gel [62].

The collapsed gels seem to be not suitable as antiseptic coatings. To avoid gel collapsing, we used CPC either in deficiency (CPC/charged gel units ratio 0.5) or in excess (CPC/charged gel units ratio 10).

### 3.2. Kinetics of the Disinfectant Release

The kinetics of CPC release from polymer coatings was studied under different conditions, using PAAm-AMPSA and PAAm-MA gels.

It is important for various practical applications to explore the release of disinfectant into small droplets of aqueous solution appearing on the surface, e.g., as a result of coughing/sneezing or touching by wet hands; therefore, to estimate the amount of CPC released, the gel samples were rinsed by a very small volume (0.2 mL) of 0.9 wt% aqueous NaCl solution. The amount of disinfectant passed into the solution at different contact times was determined by UV-spectroscopy using the absorption band of the pyridine ring of CPC at 259 nm.

**Figure 3:**
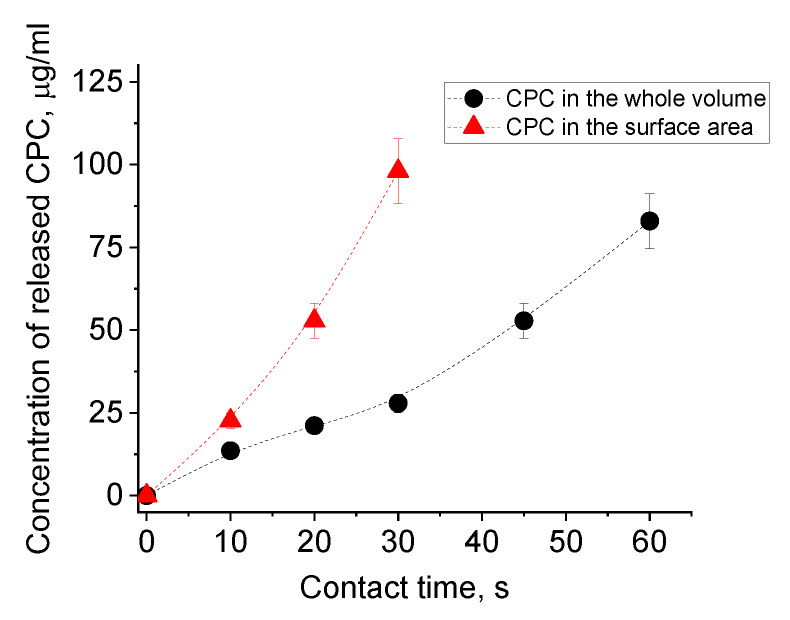
CPC release into a small volume (0.2 mL) of physiological saline from dried PAAm-AMPSA hydrogels with 10 mol% of AMPSA, which were filled with CPC by immersing them in CPC solution (PAAm-AMPSA-4v, black circles) or by applying CPC solution onto the surface of the gel (PAAm-AMPSA-4s, red triangles) at the CPC/AMPSA molar ratio equal to 0.5.

Figure 3 shows CPC release from PAAm-AMPSA-4 gels loaded with the disinfectant by whole gel immersion (PAAm-AMPSA-4v) and surface application (PAAm-AMPSA-4s). In both samples, the CPC/AMPSA molar ratio was the same (0.5). One can see that the release is much faster from PAAm-AMPSA-4s than PAAm-AMPSA-4v: the amount of CPC released in 30 s equaled, respectively, 28 µg/mL and 100 µg/mL; therefore, ‘surface incorporation’ of CPC makes it possible to increase its release from the gel significantly. This is due to the accumulation of the surfactant in the vicinity of the gel surface that was exposed to water droplets. Furthermore, the lower CPC content on the opposite surface of the hydrogel sample improves its adhesion to various surfaces, e.g., glass or polypropylene; therefore, this way of loading provides two advantages: faster CPC release and better adhesion of the polymer coating to different surfaces.

Further acceleration of CPC release can be achieved by increasing the content of CPC in the gel. All the above-described experiments were performed at the CPC/AMPSA molar ratio equal to 0.5, i.e., under the conditions of CPC deficiency relative to the number of oppositely charged polymer network units. In further studies, the CPC/AMPSA molar ratio was increased to 10. In this case, already in 2 s, the concentration of CPC in the water droplet reached 300 µg/mL, which far exceeds the concentration sufficient for complete SARS-CoV-2 inactivation (see Section 3.4). The amount of the surfactant released did not change on subsequent pouring of water onto the same place for at least 30 times during a period of 3–4 weeks; therefore, such polymer/surfactant complexes are very promising for the production of long-lasting antiseptic coatings.

One can expect that the amount of the released CPC can be finely tuned by varying other factors in addition to the initial CPC content in the gel. Those factors include characteristics of the polymer gel matrix such as the degree of swelling and the degree of cross-linking determining the gel pore size as well as the content/type of anionic groups responsible for the interaction with the disinfectant. To explore their effects on CPC release, the gel samples were immersed in a large volume of water (exceeding about 100 times that of the polymer gel) since it is difficult to measure the release profile accurately by using small water droplets.

**Figure 4:**
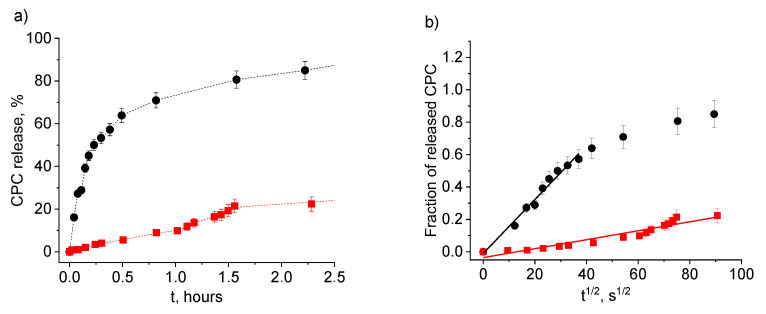
Release of CPC into physiological saline from water-swollen PAAm-AMPSA hydrogels containing 2 wt% AMPSA (PAAm-AMPSA-3v; degree of swelling, 16.0) (black circles) and 10 wt% AMPSA (PAAm-AMPSA-4v; degree of swelling, 17.1) (red squares) as a function of time t (**a**) and t^1/2^ (**b**). In both samples, the CPC/AMPSA molar ratio is 0.5, and the degree of cross-linking is 1:1000.

**Figure 5:**
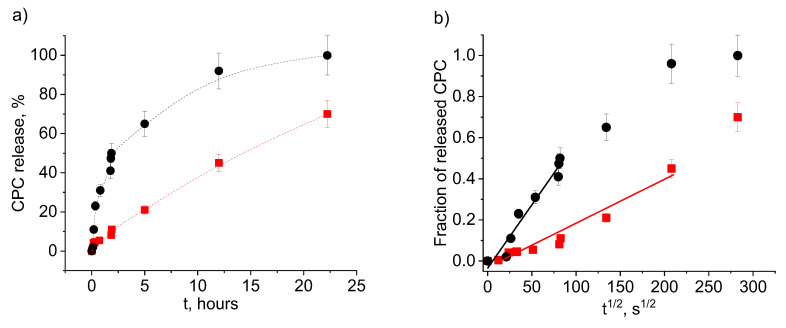
Release of CPC into physiological saline from dried PAAm-AMPSA hydrogels containing 2 wt% AMPSA (PAAm-AMPSA-3v, Table 1) (black circles) and 10 wt% AMPSA (PAAm-AMPSA-4v, Table 1) (red squares) as a function of time t (**a**) and t^1/2^ (**b**). In both samples, the CPC/AMPSA molar ratio is 0.5, and the degree of cross-linking is 1:1000.

Let us first examine the effect of the initial degree of swelling of the gel on the release of the disinfectant. The data for initially water-swollen and dried PAAm-AMPSA hydrogels are shown in Figure 4 and Figure 5, respectively. The kinetic curves of CPC release from the water-swollen hydrogel (Figure 4b) demonstrate a t^1/2^ time dependence of the fraction of the released surfactant that is characteristic of Fickian diffusion [63,64]. The approximation is valid up to approximately 60% of the released solute, which is in accordance with previously reported data for polymer systems with Fickian release behavior [63]. In Fickian behavior, the relaxation processes of the polymer are very slow in comparison to the diffusion rate of the solute inside the hydrogel [65]. Figure 5b demonstrates that CPC release from the initially dried PAAm-AMPSA hydrogel also obeys Fickian law at the early stage (up to approximately 50% of the released solute).

A comparison of Figure 4 and Figure 5 indicates that CPC release proceeds faster from the swollen as compared to the initially dried PAAm-AMPSA hydrogel. For instance, in the case of PAAm-AMPSA-3v, 50% CPC was released in 25 min and 2.2 h from the swollen and initially dried gels, respectively. This behavior is expected because the pores of the swollen gel are open for release, whereas the dried gel should first swell to make possible CPC diffusion into the outer solution. A similar effect was observed for PAAm-MA gels (Figure S3, Supplementary Materials).

**Figure 6:**
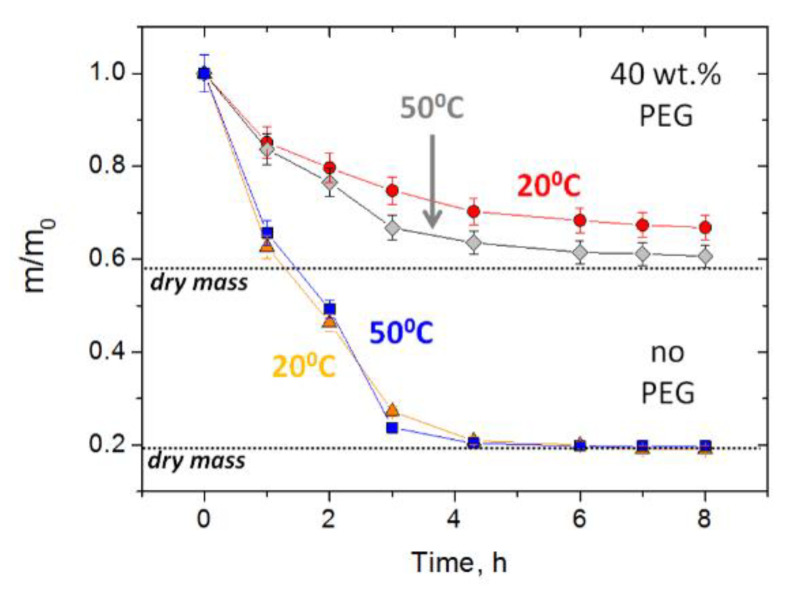
Loss of mass upon drying of PAAm-MA (10 mol%) gels containing no PEG (triangles—drying at 20 °C, squares—50 °C) or 40 wt% PEG400 (circles—drying at 20 °C, diamonds—50 °C). The initial gel samples in the form of disks with 0.5 mm thickness and 10 mm diameter were deposited on a glass substrate during drying.

To accelerate the diffusion from the dried gel, one can add hydrophilic additives (e.g., PEG) that help to retain some water in the gel matrix (Figure 6). Drying of the PAAm-MA gels with no added PEG results in an almost complete loss of water after 4 h; however, in the presence of PEG, the gels retain 25% and 20% of water after 8 h of drying at 20 °C and 50 °C, respectively. The mechanical properties of dried gel coatings were studied using a cylindrical indenter with a diameter of 8 mm, imitating finger touches (Figure S4, Supplementary Materials). It was shown that in the presence of PEG, the coating is much softer: the indentation penetration depth of 30% is achieved only at 1 N load (instead of 54 N load in the absence of PEG). The load–unload test shows an 80% reversible (elastic) deformation for PEG-containing samples and only a 5% reversible deformation for neat samples without PEG (almost fully plastic deformation). High elastic deformation of the PEG-containing coating ensures a tighter contact between the human finger and touch surface, which should increase the effectiveness of the virucidal efficacy.

**Figure 7:**
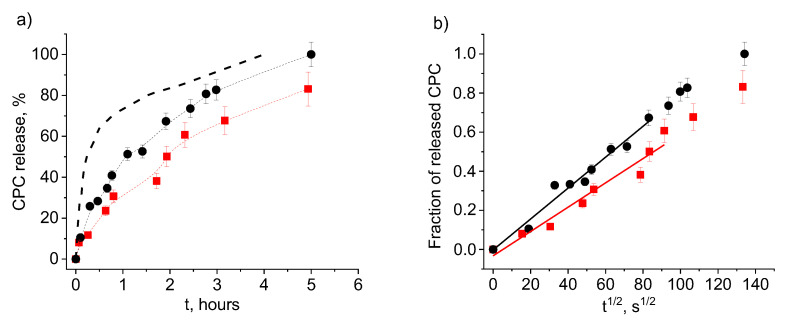
Release of CPC into physiological saline from water-swollen PAAm hydrogels with 2 wt% anionic units of different types: AMPSA (PAAm-AMPSA-6v, Table 1; degree of swelling, 5.1) (black circles) and MA (PAAm-MA, Table 1; degree of swelling, 5.0) (red squares) as a function of time t (**a**) and t^1/2^ (**b**). In both samples, the CPC/charged units molar ratio is 0.5; the degree of cross-linking is 1:500. The black dashed line describes CPC release from water-swollen PAAm-AMPSA hydrogel with 2 wt% AMPSA (PAAm-AMPSA-3v, Table 1; degree of swelling, 16.0), having a lower degree of cross-linking (1:1000).

Another parameter, which is expected to affect the rate of release, is the gel cross-linking density [66,67]. Figure 7a (full and dashed black curves) shows that increasing the degree of cross-linking slows down the rate of CPC release, as expected. Such behavior, observed previously for many hydrogels [65,67], was attributed to a smaller network mesh size and a less flexible hydrogel structure.

Let us now examine the effect of the content of anionic groups responsible for the interaction with CPC on its release. Figure 4 and Figure 5 demonstrate that, for both swollen and dried samples, the release rate depends significantly on the degree of charging of the gel (at constant CPC/AMPSA molar ratios). The greater the gel charge, the slower the release. For instance, CPC ions are completely released from more charged swollen PAAm-AMPSA gel (10 mol% AMPSA) in 12 h, whereas less charged swollen gel releases all CPC ions in 4 h; therefore, charged monomer units impede the release of oppositely charged surfactant ions from the gel. This behavior can be exploited for tuning the release time of the disinfectant in order to obtain a long-acting disinfectant coating.

Figure 7 allows us to compare CPC release profiles for hydrogels with two different types of negatively charged units (AMPSA and MA). It is seen that the release rate is somewhat lower for hydrogels with MA groups. This may indicate to stronger interaction of carboxylic groups with CPC as compared to sulfonate groups, which is consistent with a higher charge density of alkyl carbonates headgroups as compared to alkyl sulfates [68]. Previously, clear differences in the affinity of carboxylic and sulfonate groups to various cations were demonstrated both by experimental and computational results [68,69]. They were attributed to a competition for a given cation between hydrating water molecules and the anion, which is qualitatively related to charge distributions, polarizabilities, and effective field strengths of the ions [70].

Thus, the rate of the release of cationic disinfectant from oppositely charged polymer gel can be tuned in a fairly broad range by varying the concentration of CPC, the degree of swelling and the degree of cross-linking of the gel and the content/type of anionic repeat units in the polymer matrix.

### 3.3. Reloading

**Figure 8:**
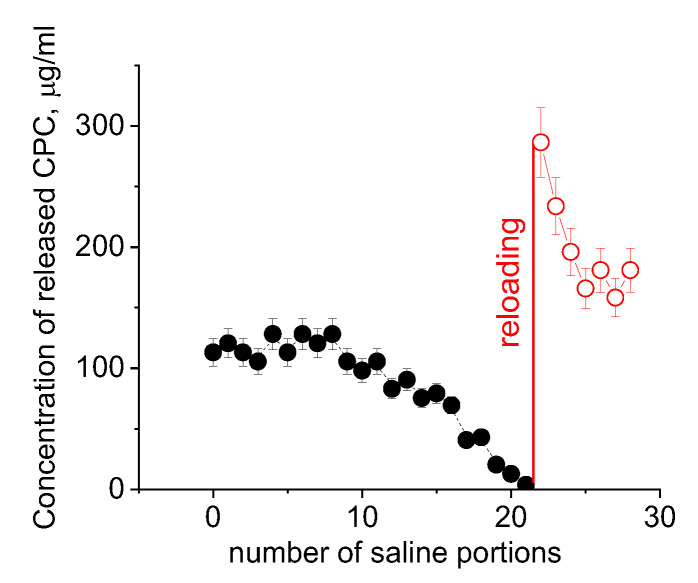
Release of CPC from dried PAAm-AMPSA-4s hydrogels to a small volume (0.2 mL) of physiological saline for original hydrogel preliminarily washed with a large volume of saline (black circles) and for reloaded hydrogel (red circles). Initial CPC/AMPSA molar ratio in the hydrogel matrix is 0.5; the CPC/AMPSA molar ratio in the hydrogel matrix upon reloading is 0.35; the solution used for reloading contained 7 wt% CPC and 25 wt% PEG.

The polyelectrolyte gel/disinfectant complexes belong to released-based [71] antiseptic materials. The lifetime of their antiseptic activity is limited by the amount of disinfectant embedded in the polymer matrix, which will exhaust over time. One can estimate that the complexes under study will keep their activity during ca. 10^3^ treatments with 0.2 mL of saline solution. Further, the polymer matrix can be refilled with the disinfectant and reused. This is illustrated in Figure 8. Since it is very time consuming to make 1000 separate treatments with 0.2 mL water aliquots to remove most of the CPC, we first removed 98% of CPC by immersing hydrogel in 200 mL of physiological saline solution, dried the hydrogel and then studied the release of the remaining disinfectant by adding many times 0.2 mL portions of saline. The kinetics of the release of disinfectant to these final volumes of salt solution is illustrated in Figure 8. When the CPC was completely removed, the polymer matrix was reloaded with the disinfectant. Figure 8 shows that the reloaded gel effectively releases the disinfectant. In this case, the first portions of released CPC contain a large amount of disinfectant, probably, because, upon reloading, some CPC molecules did not penetrate deep into the polymer matrix. To avoid this, the dried hydrogel could be treated preliminarily with water to induce gel swelling and only then treated with a CPC solution.

Thus, the polyelectrolyte gels can be reloaded with disinfectant by spraying or pouring the disinfectant solution on the gel surface.

### 3.4. Virucidal Properties

We first studied the virucidal activity of CPC in the absence of the polymer gel. Table 2 shows that CPC completely inactivates SARS-CoV-2 (IC = 100%) at concentrations equal to or higher than 0.07 mM (or 0.0024 wt%).

Table 3 shows the results of SARS-CoV-2 inactivation by CPC-containing polymer coatings. It is seen that all samples studied completely inactivate the virus (IC = 100%), reducing its titer by seven orders of magnitude. For all samples, the concentration of the released CPC exceeds that required for complete inactivation of the virus. An important point that we should pay attention to is the very short duration of the contact between the buffer solution or the virus-containing liquid with the gel coating (5 s), which was sufficient to release from the polymers an amount of CPC that ensures complete (100%) inactivation of high-titer SARS-CoV-2. There is still no exact information in the available publications regarding how many viral particles correspond to one TCID_50_. Even if we assume 10 TCID_50_ to be equivalent to as little as 2 to 4 viral particles [72], our data indicate that the surfactant released from the gel in 5 s inactivates 2 to 4 million viral particles.

Table 3 shows that the coating retains high antiviral activity for at least 7 days. Moreover, the studies of CPC release show that the amount of the surfactant released remains high enough to completely inactivate SARS-CoV-2 even in 3–4 weeks. Thus, the proposed antiseptic coatings offer significant promise as means of long-term disinfection of surfaces that can get recontaminated many times, remaining a potential source of infection.

Note that the concentrations of released CPC (Table 3) that are effective against SARS-CoV-2 are close to CPC concentrations (0.025–0.1 wt%) considered by the US Food and Drug Administration as safe when formulated in mouth rinses [73]. Several CPC toxicity studies have shown that LD_50_ of CPC is 250 mg/kg subcutaneously [73,74] and >500 mg/kg orally [75]. Percutaneous absorption of CPC is not believed to be significant [76].

Polymer matrices make it possible to tune both the concentration of the released cationic disinfectant and the rate of its release, which paves the way to finding conditions under which CPC will be delivered in desired amounts. This is very important since the use of excessive amounts of disinfectants may have a negative impact on the environment.

## 4. Conclusions

Polyelectrolyte gels with electrostatically bound disinfectant ions were demonstrated to be capable of complete and fast elimination of infectious SARS-CoV-2. The gels exhibit long-lasting antiviral activity and can be easily reloaded when the disinfectant is exhausted. When compared to common low-molecular-weight disinfectants, such materials can be regarded as safer and environmentally friendly sustainable biocides. They can deliver a high disinfectant agent concentration locally, thus minimizing the toxic effects. The coatings made from these polymer/disinfectant systems can serve to inactivate viruses in respiratory droplets appearing on surfaces (e.g., as a result of coughing or sneezing of the infected people). Such coatings can be used to cover various surfaces in public areas, such as door handles, light switches, elevator buttons, and so on. Considering that the mechanism of action of quaternary ammonium disinfectants is most likely universal, it is conceivable that the materials under study will be efficient in inactivating other enveloped viruses. Note that the proposed approach is general, and CPC should be viewed as a model disinfectant. In further studies, the same approach can be applied to other cationic disinfectants (such as BAC or double-chain surfactants such as di-N-didodecyldimethylammonium bromide [77]).

## Figures and Tables

**Table 1:** Composition of copolymer gels used for film preparation.

Samples	Total Concentration of Monomers, M	Fraction of Charged Monomer Units		Concentration of Cross- Linker, M	Degree of Cross- Linking	Concentration of PEG, wt%
		Feed	^1^NMR			
PAAm-AMPSA-1v	1.4	0.02	0.019	0.00175	1:800	10
PAAm-AMPSA-1s	1.4	0.02	0.020	0.00175	1:800	10
PAAm-AMPSA-2s	1.4	0.02	0.018	0.00175	1:800	20
PAAm-AMPSA-3v	2.8	0.02	0.020	0.00280	1:1000	0
PAAm-AMPSA-4v	2.8	0.10	0.091	0.00280	1:1000	0
PAAm-AMPSA-5v	2.8	0.02	0.018	0.00560	1:500	0
PAAm-MA	2.8	0.02	0.017	0.00560	1:500	0
PVP-MA	2.8	0.10	0.30	0.014	1:200	0

**Table 2:** SARS-CoV-2 inactivation by CPC solution (contact time, 1h).

Concentration		Virus Titer			Inhibition Coefficient IC, %
mM	wt%	Control A_c_	Experiment A_e_	Log10 Reduction A	
0.28	0.0095	7.0	0	7.0	100
0.14	0.0048	7.0	0	7.0	100
0.07	0.0024	7.0	0	7.0	100
0.025	0.0009	0	7.0	0	0

**Table 3:** SARS-CoV-2 inactivation by antiseptic polymer gels (contact time, 5 s).

Sample	Experimental Conditions	Initial CPC Concentration in the Gel, wt%	Concentration of Released CPC, wt%	Virus Titer			Inhibition Coefficient IC, %
				Control A_c_	Experiment A_e_	Log10 Reduction A	
PAAm-AMPSA-1v	Rinsing *, day 1	8.3	0.057	7.00	0.00	7.00	100
PAAm-AMPSA-1s	Rinsing *, day 1	2.0	0.100	7.00	0.00	7.00	100
PAAm-AMPSA-2s	Rinsing *, day 1	2.0	0.145	7.00	0.00	7.00	100
PVP-MA-v	Rinsing *, day 1	2.0	0.078	7.00	0.00	7.00	100
PAAm-AMPSA-2s	Rinsing *, day 7	2.0	0.100	7.00	0.00	7.00	100
PVP-MA-v	Rinsing *, day 7	2.0	0.016	7.00	0.00	7.00	100
PAAm-AMPSA-1v	Direct contact **	8.3	-	7.00	0.00	7.00	100
PVP-MA-v	Direct contact **	2.0	-	7.00	0.00	7.00	100

* Polymer gels were rinsed by applying dropwise 450 µL physiological saline onto their surface and collecting the flowing liquid in 5 s. Rinsing was performed on day 1 or day 7 after preparation of the gel sample. ** Direct contact of polymer gels with virus-containing liquid for 5 s.

## Data Availability

The data presented in this study are openly available.

## Supplementary Materials

The following supporting information can be downloaded at: https://www.mdpi.com/article/10.3390/polym14122444/s1, Figure S1: Absorption spectra of CPC solutions at 0.24 mmol/L and 0.075 mmol/L; Figure S2: Dependence of the intensity of the pyridinium ring absorbance peak at 259 nm on CPC concentration; Figure S3: Release of CPC into physiological saline from water-swollen and dried PAAm-MA hydrogels containing 2 mol% MA units (sample PAAm-MA, Table 1) as a function of time t (a) and t^1/2^ (b); Figure S4: Panel (a): Load-unload test (a force as a function of indentation depth) for PAAm-AMPSA-4s hydrogels without PEG and with 22 wt% PEG. Panel (b): Part of the graph, shown as a dashed rectangle in panel (a), enlarged for clarity; Table S1: Cytotoxicity of cetylpyridinium chloride (CPC) and benzalkonium chloride (BAC) to Vero E6 cells. References [78,79,80,81] are cited in the Supplementary Materials.
