# Supplementary material for: Antiseptic Polymer–Surfactant Complexes with Long-Lasting Activity against SARS-CoV-2
Source: Polymers (Basel). 2022 Jun 16;14(12):2444. doi: 10.3390/polym14122444 (PMC9228194; doi:10.3390/polym14122444)
Supplement: Supplementary file 1 [file polymers-14-02444-s001.zip › polymers-1764065-supplementary.pdf]

Supplementary Materials

for

Antiseptic Polymer-Surfactant Complexes with Long-Lasting Activity Against SARS-CoV-2

Vyacheslav S. Molchanov [1], Andrey V. Shibaev [1], Eduard V. Karamov [2,3], Viktor F. Larichev [2], Galina V. Kornilaeva [2], Irina T. Fedyakina [2], Ali S. Turgiev [2,3], Olga E. Philippova [1,*] and Alexei R. Khokhlov [1]

[1] Physics Department, Lomonosov Moscow State University, 119991 Moscow, Russia; molchanov@polly.phys.msu.ru (V.S.M.); shibaev@polly.phys.msu.ru (A.V.S.); khokhlov@polly.phys.msu.ru (A.R.K.)
[2] Gamaleya National Research Center for Epidemiology and Microbiology of the Russian Ministry of Health, 123098 Moscow, Russia; karamov2004@yandex.ru (E.V.K.); vlaritchev@mail.ru (V.F.L.); kornilaeva@yandex.ru (G.V.K.); irfed2@mail.ru (I.T.F.); turgiev@ld.ru (A.S.T.)
[3] National Medical Research Center of Phthisiopulmonology and Infectious Diseases of the Russian Ministry of Health, 127473 Moscow, Russia
Correspondence: phil@polly.phys.msu.ru (O.E.P.)

1. UV-Vis Spectroscopy Studies

Absorption spectra were measured for certain solutions of CPC to determine the concentration of CPC released from hydrogels.

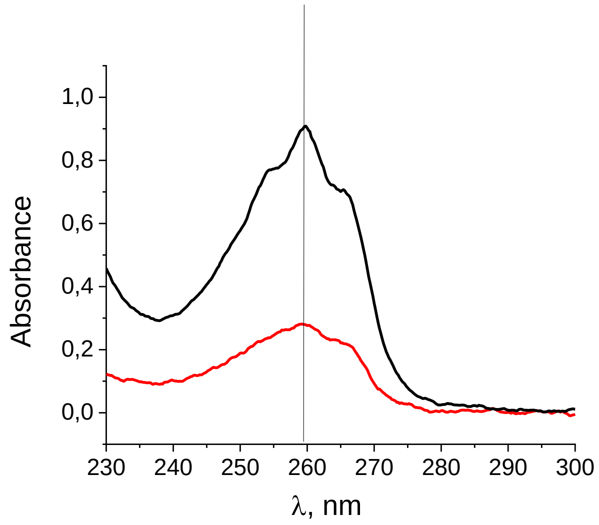

Figure S1. Absorption spectra of 0.24 mmol/L (black curve) and 0.075 mmol/L (red curve) CPC solutions.

The concentration dependence of the intensity of peak at 259 nm has been plotted to determine the extinction coefficient from the slope.

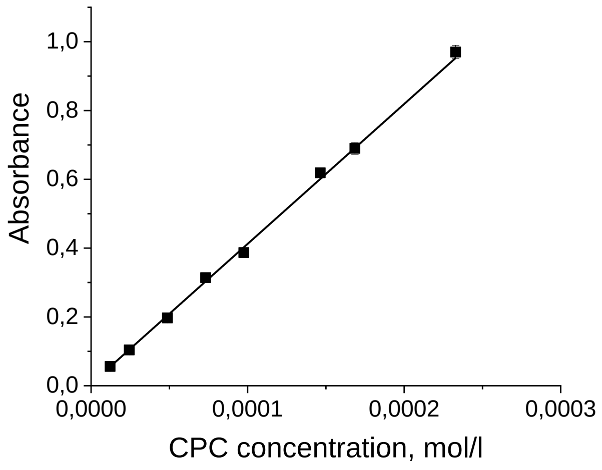

Figure S2. Dependence of the intensity of the pyridinium ring absorbance peak at 259 nm on CPC concentration.

2. Kinetics of CPC Release from PAAm-MA hydrogels

The kinetics of CPC release from polymer coatings was studied for water-swollen and dried PAAm-MA hydrogels. It follows from the kinetic curves (Figure S3b) that the dependence of the fraction of the released CPC on time ($t^{1/2}$) is characteristic of Fickian diffusion mechanism of the release.

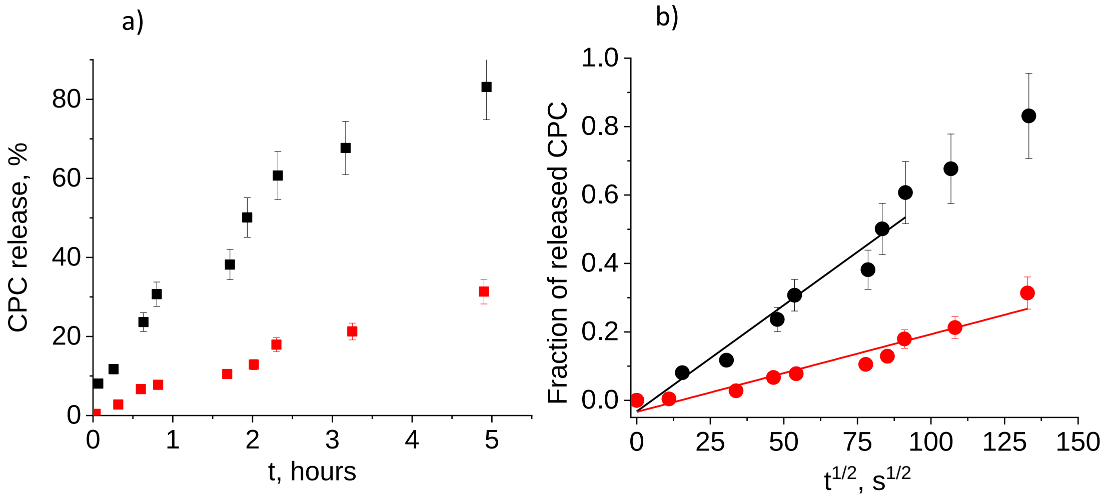

Figure S3. Release of CPC into physiological saline from water-swollen (black symbols) and dried (red symbols) PAAm-MA hydrogels containing 2 mol% MA units (sample PAAm-MA, Table 1) as a function of time t (a) and $t^{1/2}$ (b). In both samples the CPC/MA molar ratio is 0.5, the degree of cross-linking is 1:500.

3. Mechanical Properties of the Hydrogels

The mechanical properties of the hydrogel films with and without added PEG were studied on a testing machine using a cylindrical indenter with a diameter of 8 mm. In these experiments, the loading force was measured as a function of the indentation depth (Figure S4). This makes it possible to estimate the resistance of a material to the indentation by an indenting probe.

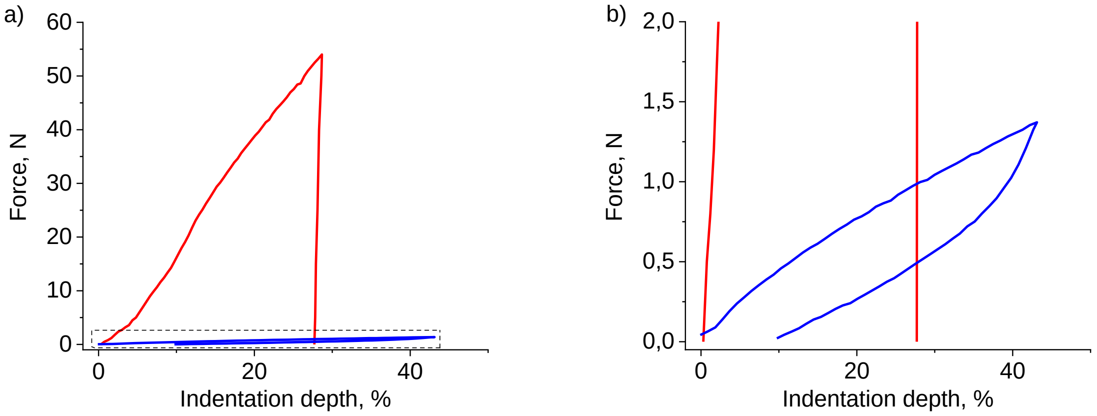

Figure S4. Panel (**a**): Load-unload test (a force as a function of indentation depth) for PAAm-AMPSA-4s hydrogels without PEG (red curve) and with 22 wt% PEG (blue curve). Panel (**b**): Part of the graph, shown as a dashed rectangle in panel (a), enlarged for clarity.

4. Cytotoxicity Tests

The efficacy of antiseptics and disinfectants is assessed primarily by their ability to inactivate (lyse, otherwise damage or render functionally disabled) an infectious agent in the absence of or prior to the contact with its target cell. Conversely, the effects of drugs (for chemoprevention / chemotherapy) on an infectious agent are (1) always set against cytotoxicity and (2) often cannot be studied outside its target cell. Although the setup of experiments assessing the virucidal activity of CPC ruled out any effect on Vero E6 cells, we determined its median cytotoxic concentration (CC_{50}) for two exposure times. Benzalkonium chloride (BAC) was tested in parallel as a reference disinfectant / antiseptic.

Table S1. Cytotoxicity of cetylpyridinium chloride (CPC) and benzalkonium chloride (BAC) to Vero E6 cells

Disinfectant / antiseptic	*CC_{50}*			
	72 h		*96 h*	
	mM	*wt %*	*mM*	*wt %*
Benzalkonium chloride	0.299	0.01025	0.022	0.00075
Cetylpyridinium chloride	0.098	0.00336	0.013	0.00045

In brief, serial dilutions of CPC and BAC in support medium (DMEM, 2% FCS) were added to confluent Vero E6 monolayers in 96-well plates, and the plates were incubated at 37°C in 5% CO_2 for 72 or 96 h. Each dilution was tested in four replicates; eight wells were used as a vehicle control. Cell viability was assessed by the MTS test (CellTiter 96® AQueous One Solution Cell Proliferation Assay; Promega, Madison, USA) based on the ability of live cells to convert 3-(4,5-dimethylthiazol-2-yl)-5-(3-carboxymethoxyphenyl)-2-(4-sulfophenyl)-2H-tetrazolium, inner salt (MTS) into a colored formazan product that is soluble in tissue culture medium [1,2]. When the incubation was completed, the culture medium was removed from the wells, and 100 μL of support medium (DMEM, 2% FCS) and 20 μL MTS reagent were added to each well, and the plate was incubated at 37°C for additional 3 h. Absorbance was measured at 490 nm on an iMark plate reader (Bio-Rad Laboratories, Hercules, USA) using 630 nm as a reference wavelength. The values of CC_{50} were derived from viability dependences on surfactant concentrations, using non-linear regression analysis (Prism 6; GraphPad Software, San Diego, USA).

The comparison of data in Table 2 and Table S1 demonstrates that SARS-CoV-2 exposed to 0.07 mM CPC for 1 h is completely inactivated, whereas Vero E6 cells retain at least 50% viability on exposure to a higher concentration (0.098 mM) for 72 h. As follows from Table S1, the values of CC_{50} are strongly exposure-dependent: disinfectant cytotoxicity to Vero E6 cells increases with the incubation time (and, correspondingly, CC_{50} values become lower). A similar observation has been reported for cultured human periodontal ligament cells exposed to dental antiseptics for 10 s, 20 s, 30 s, 10 min, 20 min, 30 min, 24 h, and

48 h [3]. Since studies of CPC cytotoxicity were beyond the scope of this work, we did not measure CC_{50} values at other incubation times. In fact, CPC is widely present in personal care products (mouthwashes, cough lozenges, cleaning agents); it is also being used as an antimicrobial agent for meat and poultry products (up to 1%) and has been used in multiple clinical trials, including as a treatment against respiratory infections [4].

References

1. Barltrop, J.A.; Owen, T.C.; Cory, A.H.; Cory, J.G. 5-(3-carboxymethoxyphenyl)-2-(4,5-dimethylthiazolyl)-3-(4-sulfophenyl)tetrazolium, inner salt (MTS) and related analogs of 3-(4,5-dimethylthiazolyl)-2,5-diphenyltetrazolium bromide (MTT) reducing to purple water-soluble formazans as cell-viability indicators. *Bioorg. Med. Chem. Lett*. **1991**, *1*(11), 611-614. https://doi.org/10.1016/S0960-894X(01)81162-8.

2. Berridge, M.V.; Tan, A.S. Characterization of the cellular reduction of 3-(4,5-dimethylthiazol-2-yl)-2,5-diphenyltetrazolium bromide (MTT): subcellular localization, substrate dependence, and involvement of mitochondrial electron transport in MTT reduction. *Arch. Biochem. Biophys*. **1993**, *303*(2), 474-82. https://doi.org/10.1006/abbi.1993.1311.

3. Khorolsuren, Z.; Lang, O.; Vag, J.; Kohidai, L. Effect of dental antiseptic agents on the viability of human periodontal ligament cells. *Saudi Dent. J*. **2021**, *33*(8), 904-911. https://doi.org/10.1016/j.sdentj.2021.09.016.

4. Mukherjee, P.K.; Esper, F.; Buchheit, K.; Arters, K.; Adkins, I.; Ghannoum, M.A.; Salata, R.A. Randomized, double-blind, placebo-controlled clinical trial to assess the safety and effectiveness of a novel dual-action oral topical formulation against upper respiratory infections. *BMC Infect. Dis*. **2017**, *17*(1), 74. https://doi.org/10.1186/s12879-016-2177-8
